# Outcome prediction based on [18F]FDG PET/CT in patients with pleural mesothelioma treated with ipilimumab and nivolumab +/- UV1 telomerase vaccine

**DOI:** 10.1007/s00259-024-06853-0

**Published:** 2024-08-12

**Authors:** Solfrid Thunold, Eivor Hernes, Saima Farooqi, Åsa Kristina Öjlert, Roslyn J. Francis, Anna K. Nowak, Weronika Maria Szejniuk, Søren Steen Nielsen, Susana Cedres, Marc Simo Perdigo, Jens Benn Sørensen, Carin Meltzer, Lars Tore Gyland Mikalsen, Åslaug Helland, Eirik Malinen, Vilde Drageset Haakensen

**Affiliations:** 1 Dept of Oncology, Oslo University Hospital, Oslo, Norway; 2 Dept of Cancer Genetics, Institute for Cancer Research, Oslo University Hospital, Oslo, Norway; 3 Division of Radiology and Nuclear Medicine, Oslo University Hospital, Oslo, Norway; 4 Institute of Clinical Medicine, Faculty of Medicine, University of Oslo, Oslo, Norway; 5 Dept of Nuclear Medicine, Sir Charles Gairdner Hospital, Perth, Australia; 6 Medical School of The University of Western Australia, Perth, Australia; 7 National Centre for Asbestos-Related Diseases, University of Western Australia, Perth, Australia; 8 Medical Oncology, Sir Charles Gairdner Hospital, Perth, Australia; 9 Department of Nuclear Medicine, Aalborg University Hospital, Aalborg, Denmark; 10 Dept of Nuclear Medicine, Hospital Universitari Vall d’Hebron, Barcelona, Spain; 11 Clinical Cancer Research Center & Department of Oncology, Aalborg University Hospital, Aalborg, Denmark; 12 Department of Clinical Medicine, Aalborg University, Aalborg, Denmark; 13 Vall d’Hebron Institute of Oncology, Hospital Universitari Vall d’Hebron, Barcelona, Spain; 14 Department of Oncology, Rigshospitalet, Copenhagen University Hospital, Copenhagen, Denmark; 15 Department of Clinical Medicine, University of Copenhagen, Copenhagen, Denmark; 16 Department of Physics and Computational Radiology, Division of Radiology and Nuclear Medicine, Oslo University Hospital, Oslo, Norway; 17 Department of Life Sciences and Health, Oslo Metropolitan University, Oslo, Norway; 18 Dept of Radiation Biology, Institute for Cancer Research, Oslo University Hospital, Oslo, Norway; 19 Department of Physics, University of Oslo, Oslo, Norway

**Keywords:** [18F]FDG PET, Immunotherapy, Pleural mesothelioma, Telomerase vaccine, SUV, MTV

## Abstract

**Purpose:**

The introduction of immunotherapy in pleural mesothelioma (PM) has highlighted the need for effective outcome predictors. This study explores the role of [18F]FDG PET/CT in predicting outcomes in PM treated with immunotherapy.

**Methods:**

Patients from the NIPU trial, receiving ipilimumab and nivolumab +/- telomerase vaccine in second-line, were included. [18F]FDG PET/CT was obtained at baseline (n = 100) and at week-5 (n = 76). Metabolic tumour volume (MTV) and peak standardised uptake value (SUV_peak_) were evaluated in relation to survival outcomes. Wilcoxon rank-sum test was used to assess differences in MTV, total lesion glycolysis (TLG), maximum standardised uptake value (SUV_max_) and SUV_peak_ between patients exhibiting an objective response, defined as either partial response or complete response according to the modified Response Criteria in Solid Tumours (mRECIST) and immune RECIST (iRECIST), and non-responders, defined as either stable disease or progressive disease as their best overall response.

**Results:**

Univariate Cox regression revealed significant associations of MTV with OS (HR 1.36, CI: 1.14, 1.62, p < 0.001) and PFS (HR 1.18, CI: 1.03, 1.34, p = 0.02), while multivariate analysis showed a significant association with OS only (HR 1.35, CI: 1.09, 1.68, p = 0.007). While SUV_peak_ was not significantly associated with OS or PFS in univariate analyses, it was significantly associated with OS in multivariate analysis (HR 0.43, CI: 0.23, 0.80, p = 0.008). Objective responders had significant reductions in TLG, SUV_max_ and SUV_peak_ at week-5.

**Conclusion:**

MTV provides prognostic value in PM treated with immunotherapy. High SUV_peak_ was not associated with inferior outcomes, which could be attributed to the distinct mechanisms of immunotherapy. Early reductions in PET metrics correlated with treatment response.

**Study registration:**

The NIPU trial (NCT04300244) is registered at clinicaltrials.gov. https://classic.clinicaltrials.gov/ct2/show/NCT04300244?cond=Pleural+Mesothelioma&cntry=NO&draw=2&rank=4

**Supplementary Information:**

The online version contains supplementary material available at 10.1007/s00259-024-06853-0.

## Background

Pleural Mesothelioma (PM) has a dismal prognosis, with a median overall survival of approximately one year [1]. Given the large individual variations in disease progression and the scarcity of prognostic and predictive biomarkers, selecting the most effective therapeutic approach is difficult. Although new therapeutic modalities have been introduced [2], predicting which patients will respond to treatment remains a challenge.

In the context of multimodal treatment, comprising chemotherapy, surgery and possibly radiation therapy, [^18^F]fluorodeoxyglucose positron emission tomography/computed tomography ([^18^F]FDG PET/CT) serves as a standard assessment tool for evaluating disease extension. For inoperable patients, it is not routinely used [3–5].

[^18^F]FDG PET features have shown prognostic potential for malignancies such as colorectal cancer, head and neck cancer, lymphomas, and lung cancer [6–9]. Although research has highlighted the potential of both volumetric PET features, including metabolic tumour volume (MTV), and maximum standardised uptake value (SUV_max_) for PM summarised in some recent reviews and meta-analyses [10–12], existing studies often suffer from limitations such as small sample sizes or retrospective designs. A few studies suggest that PET may be superior to CT for response assessment in PM [13–16], which becomes increasingly relevant with the advent of immunotherapy [2].

The introduction of immunotherapy has revealed several unique tumour response patterns, some of which may be difficult to detect using standard radiological imaging due to their lack of volumetric changes [17–19]. Moreover, differentiating between progressive disease and pseudoprogression poses a challenge. The ability of [^18^F]FDG PET/CT to depict the metabolic microenvironment, in addition to anatomical structures, may be beneficial in evaluating early response to immunotherapy since the functional response to immunotherapy usually precedes radiological response [17–21].

Thus, further research is needed to evaluate the effectiveness of 18F-FDG PET/CT in immunotherapy response assessment for PM.

To our knowledge, this is the first prospective, multicentre trial on [^18^F]FDG PET/CT in PM treated with double immunotherapy comparing baseline to follow-up at week-5. Our study aimed to evaluate PET as a tool for predicting outcomes and assessing early response in patients with PM treated with immunotherapy.

## Method

### Patient population

The NIPU trial (NCT04300244) is a phase II, randomised, open-label, multicentre study that evaluates nivolumab and ipilimumab with or without UV1 vaccination as second-line treatment in patients with PM [22]. Patients with progressive disease after first-line platinum doublet were eligible given good performance status and acceptable organ function. Patients were randomised 1:1 to ipilimumab and nivolumab alone or in combination with the UV1 telomerase peptide vaccine (16). Nivolumab (240 mg) was administered intravenously every two weeks, and ipilimumab (1mg/kg) every six weeks, until disease progression, intolerable side effects, or for a maximum of 24 months. Additionally, patients in arm A received the UV1 vaccine regimen: three vaccinations in the first week, one in the second week, and four more over the following 11 weeks, totalling eight vaccinations in 13 weeks (21).

### Ethics

The trial was approved by the regional ethics committee (20/47804) and each sites ethics committee and was conducted in accordance with the Declaration of Helsinki of the World Medical Association and ICH E6 for Good Clinical Practice. All patients provided written informed consent.

### PET scans

**Fig. 1:**
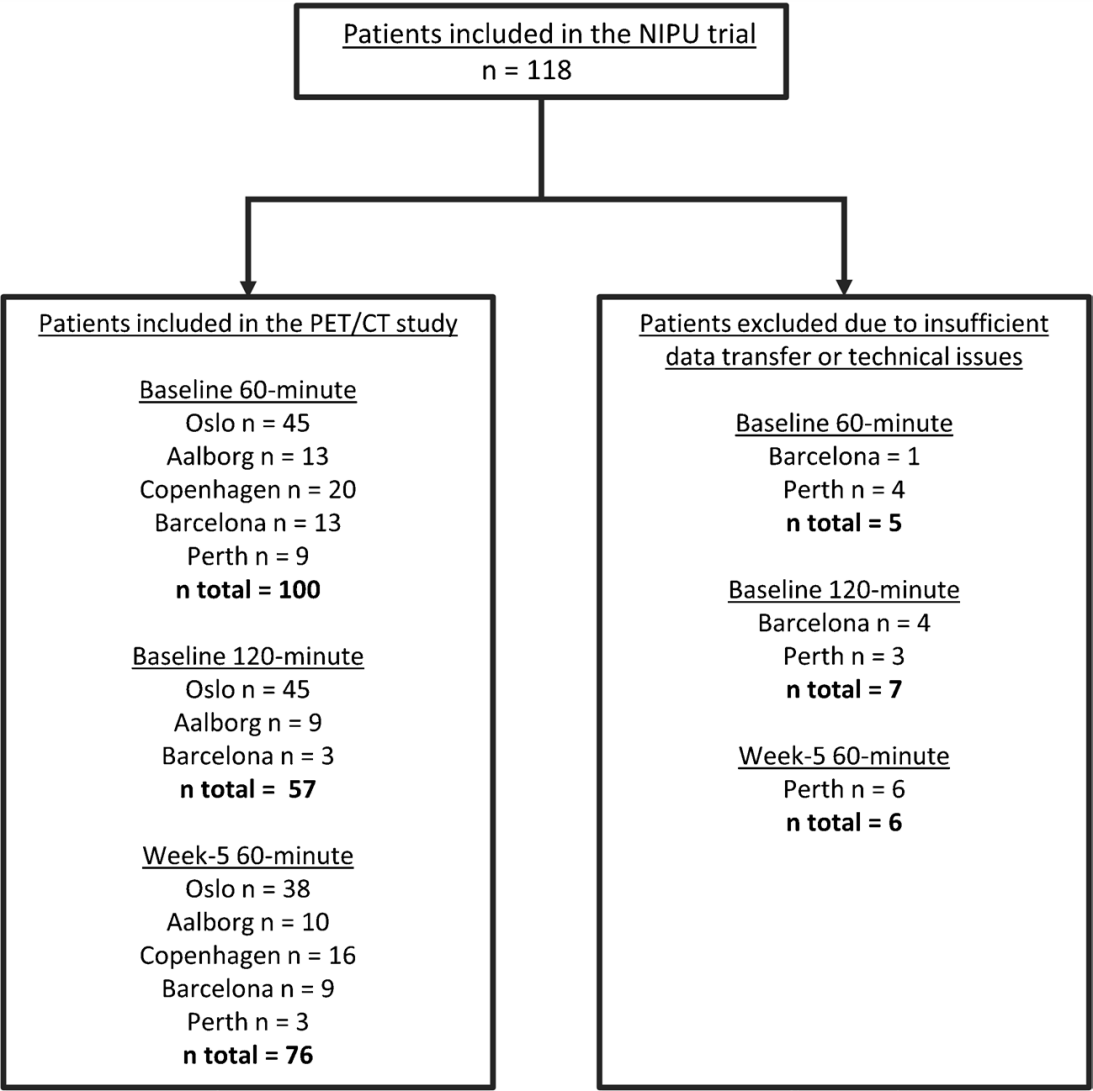
Flow chart illustrating the number of patients who underwent [^18^F]FDG PET/CT scans at baseline and week-5. [^18^F]FDG PET/CT = [^18^F]fluorodeoxyglucose positron emission tomography/computed tomography. Patients excluded were those with insufficient image data transfer or technical issues with the transferred image data

Each patient underwent [^18^F]FDG PET/CT at baseline, five weeks after the start of the treatment (week-5), one year after randomisation or at the time of progression. The PET/CT was conducted 60 minutes post-18F-FDG injection after a fasting period of six hours. A sub-cohort of the patients, predefined by the study site, underwent an additional scan 120 minutes post-injection, referred to as a late-phase scan. Fig. 1.

PET scans conducted at the time of progression or after one year were limited to a fraction of the patients, as determined by the investigator. These scans have not been included in the analyses presented in this article. Patients were injected with 3-3.7 MBq/kg [18F]FDG. Images were acquired using hybrid PET/CT systems, including Siemens Biograph 40 mCT, Siemens Biograph 64 Vision 600, and Siemens Biograph 64 mCT models. The standard 60-minute scans captured images from the base of the skull to the thighs using 2 minutes/bed or a scan speed of 0.6-1.5 mm/s depending on system. The extended 120-minute scans included only the thoracic region using 2-3 minutes/bed or a scan speed of 0.6-1 mm/s. See Table 1 for details. Patients from the sites in Oslo (Norway), Aalborg (Denmark), Copenhagen (Denmark), Barcelona (Spain) and Perth (Australia) were included in the analyses. Scans from two of the study sites (Oslo and Aalborg) were acquired on systems accredited by the European Association of Nuclear Medicine Research Ltd (EARL) harmonisation programme and reconstructed to meet the EARL Standards 2 specifications [23]. Image data were collected and stored in TSD, a secure transfer and storage system at the University of Oslo, Norway. The PET image analyses were centralised and conducted at the main study site in Oslo.

**Table 1:** PET protocol at the different study sites. ^a, b^ Patients from Oslo and Aalborg met the EARL2 specifications, except for two patients from Aalborg (baseline 60-minute scan), four patients from Oslo (two baseline 60-minute, four baseline 120-minute and one week-5 60-minute scans), where a Gauss filter of 2 mm was used. EARL = European Association of Nuclear Medicine Research Ltd

Study site	Oslo	Aalborg	Copenhagen	Perth	Barcelona
Number of patients	45	13	20	9	13
Manufactor	Siemens	Siemens	Siemens	Siemens	Siemens
Model name	Biograph mCT 40	Biograph mCT 64	Biograph 64 Vision	Biograph mCT64	Biograph mCT 64
EARL2 compliant	Yes^a^	Yes^b^	No	No	No
MBq/kg	3	3.5	3.5	3	3.7
Scan speed 60 min	2 min/bed	0.6-1 mm/sec	1.5 mm/sec	2 min/bed	2 min/bed
Minutes/bed 120 min	3 min/bed	0.6-1 mm/sec	-	3 min/bed	2 min/bed
TOF	Yes	Yes	Yes	Yes	Yes
PSF	Yes	Yes	Yes	Yes	Yes
Iterations and subsets	2i21s	2i21s	4i5s	2i21s	3i21s
Gauss filter	5 mm	4 mm	2 mm	5-6 mm	2 mm
Matrix size	400 x 400	400 x 400	440 x 440	200 x 200	200 x 200
Pixel spacing/slice thickness (mm)	2.04/2.04/3.00	2.04/2.04/5.00	1.65/1.65/2.00	4.07/4.07/5.00	4.07/4.07/1.50

Tumour lesions were identified according to the PET Response Criteria for Solid Tumours (PERCIST 1.0) [24] using Syngo.via, *Lesion Scout with Auto-ID version VB60A (Siemens Healthcare)* [25]. The software automatically identifies lesions, based on an aorta region input and a percentage threshold relative to SUV_max_, filtering out normal tissue as recognised by the software. The threshold was manually adjusted for each image to obtain segmentations visually corresponding to the lesion as observed in a 0-10 SUV window, through consensus by two investigators (E.H., S.T.). Segmentations less than 0.5 ml were discarded. Lesions erroneously flagged as normal tissue were included, and vice versa. The use of fixed percentage thresholds, fixed SUV, and thresholds based on blood background were initially attempted but did not provide satisfactory delineation, see supplementary (Fig. S1). The software computed MTV (in units of cm^3^), total lesion glycolysis (TLG), SUV_max_ and peak standardised uptake value (SUV_peak_) for each lesion. Separate lesions outside the hemithorax were not included. The highest SUV_max_ and SUV_peak_ values and the sum of all segmented lesions for MTV and TLG were analysed. TLG was defined as MTV x mean standardised uptake value (SUV_mean_), SUV_max_ as the pixel exhibiting the highest uptake of [18F]FDG, and SUV_peak_ as the 1 cm^3^ volume with the highest [18F]FDG uptake within the tumour.

### Radiological imaging and response assessment

A CT with intravenous contrast was performed at baseline and every 6 weeks for the first 12 months following randomisation (± 7 days) and every 12 weeks thereafter (± 7 days), with additional scans performed as clinically indicated. Radiological assessment was performed according to modified Response Criteria in Solid Tumours (mRECIST) [26]. If patients were assessed as being in complete response (CR), partial response (PR) or stable disease (SD) following an earlier assessment as progressive disease (PD), they were categorised as CR, PR or SD in accordance with immune RECIST (iRECIST) [27]. Patients were classified as `objective responders` if their best overall response was either CR or PR. Conversely, those who exhibited either SD or PD as their best overall response were categorised as 'non-responders`.

### Statistics

Statistical analyses were conducted using R version 4.2.2 (2022-10-31). Non-parametric statistical tests were preferred because of the skewed distribution of the data. For analyses requiring normal distribution, a logarithmic transformation was performed. To analyse survival outcomes, Kaplan-Meier plots with log-rank test was performed. Patients were divided into two subgroups based on values above or below the median MTV or the median SUV_peak_ derived from both the standard 60-minute and the 120-minute scans. These subgroups were referred to as ‘low MTV’ and ‘high MTV’, and ‘low SUV_peak_’ and ‘high SUV_peak_’, respectively. Progression-free survival (PFS) was calculated as time from the start of the treatment to the time of PD or death from any cause. Overall survival (OS) was calculated as time from the start of the treatment to death from any cause. Univariate and multivariate Cox regression models were used to assess the associations between PET features and PFS and OS. Spearman correlation was performed to assess the correlation between the covariates in the Cox-model (Supplementary Fig. S2). Due to a high correlation between MTV and TLG, and SUV_max_ and SUV_peak_, respectively, TLG and SUV_max_ were not included in the multivariate analyses. In addition to treatment arm (+/- UV1 vaccine), Eastern Cooperative Oncology Group (ECOG) performance status, histology and neutrophil-to-lymphocyte-ratio (NLR) were included as covariates in the multivariate analyses, as they have shown to be of prognostic value in PM [28–30]. In Cox regression analyses, the hazard ratios (HRs) and their corresponding confidence intervals (CIs) for continuous covariates are presented as log-transformed values. Wilcoxon rank-sum test was conducted to compare differences in baseline PET features and the changes in PET features from baseline to week-5 between objective responders and non-responders. Additionally, the Wilcoxon test was performed to compare differences in PET features between patients who were 'programmed death ligand-1 (PD-L1) positive' and those who were PD-L1 negative. A significance level of 0.05 was established for testing of predictive power. Confidence intervals (CI) were at the 95% level. To ensure data quality, all analyses were repeated using only the patients with scans accredited by EARL2.

## Results

A total of 118 patients were included in the trial, 59 in each arm. The baseline PET scans of 100 patients were included in the analysis, of whom 57 had both 60-and 120-minute baseline scans. 76 of the patients underwent an interim PET scan at week-5 (Fig. 1).

Among the 100 patients included in the PET analysis, the best overall response observed was PR in 18 patients (18 %), SD in 53 patients (53 %) and PD in 25 patients (25 %). None of the patients achieved CR. Four patients did not undergo radiological response assessment as deteriorating conditions and death prevented a follow-up CT scan. Of the 18 patients with PR, 14 had a PET scan at week-5. Among these 14 patients, seven already showed PR at the first CT response assessment (week 5/6). See Table 2 for patient characteristics.

**Table 2:** Characteristics of the patients included in the analysis. PET (positron emission tomography) features from the baseline 60-minute scan. UV1 = UV1 telomerase vaccine. ECOG = Eastern Cooperative Oncology Group performance status. Best overall response according to mRECIST (modified Response Criteria in Solid Tumours) and iRECIST (immune RECIST). MTV = metabolic tumour volume. TLG = total lesion glycolysis. SUVmax = maximum standardised uptake value. SUVpeak = peak standardised uptake value. NLR = neutrophil-to-lymphocyte-ratio

Patient characteristic	N= 100^1^
Age	71 (64, 75)
Gender	
Female	23 (23%)
Male	77 (77%)
Treatment alarm	
+UVI	49 (49%)
-UVI	51 (51%)
ECOG	
ECOG 0	30 (30%)
ECOG 1	70 (70%)
PD-L1	
PD-L1 negative	55 (55%)
PD-L1 positive	14 (14%)
Unknown	31 (31%)
Histology	
Epitheloid	77 (80%)
Non-epitheloid	19 (20%)
Unknown	4
Best overall response	
Progressive disease	25 (25%)
Stable disease	53 (53%)
Partial response	18 (18%)
Unknown	4 (4%)
MTV (cm^3^)	196 (44, 377)
Unknown	1
TLG	839 (227, 1916)
Unknown	1
SUV_max_	14 (10, 17)
SUV_peak_	9.6 (6.7, 13.1)
NLR	3.3 (2.1, 5.2)
Unknown	3

^1^Median (OQR; n (%)

SUV_max_ and SUV_peak_ were obtained for all patients, however, MTV and TLG are unknown in one patient where a satisfactory volume delineation was not feasible at the standard 60-minute baseline scan and week-5 scan because of a low tumour-to-surrounding-tissue ratio.

**Fig. 2:**
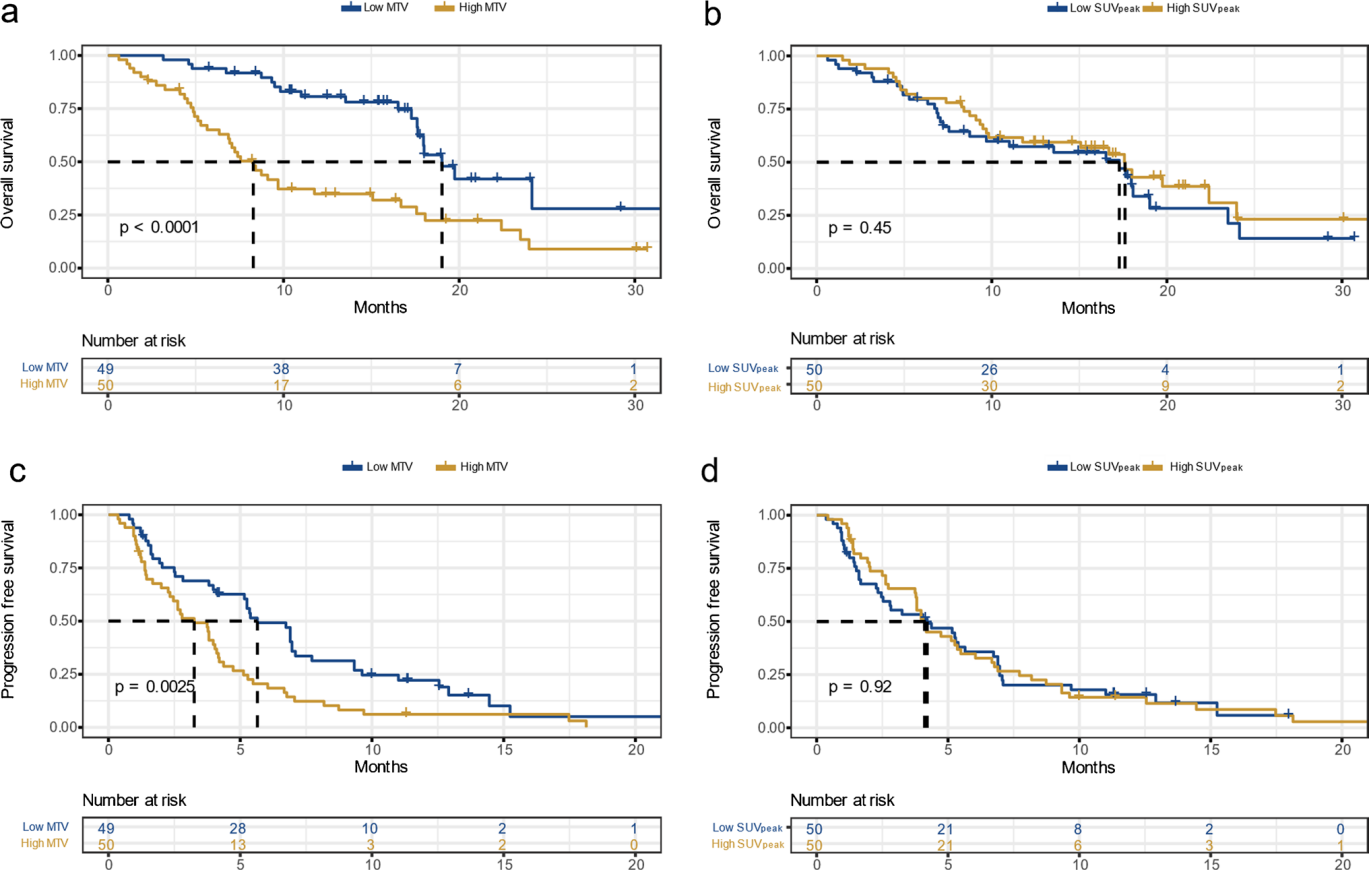
Kaplan Meyer curves with log-rank test based on MTV and SUV_peak_ from the baseline 60-minute scan. Panels **a**) and **c**) illustrate overall survival (OS) and progression free survival (PFS), respectively, where Low MTV and High MTV are grouped based on values below or above the median MTV. Panels **b**) and **d**) illustrate OS and PFS, respectively, where Low SUV_peak_ and High SUV_peak_ are grouped based on values below or above the median SUV_peak_. MTV = metabolic tumour volume. SUV_peak_ = peak standardised uptake value

The groups with low MTV from both the baseline 60-minute and 120-minute scans had significantly better OS and PFS than the groups with high MTV (Fig. 2 and Supplementary Fig. S4)*.* No significant difference in OS or PFS was found between groups with low or high baseline 60-minute and 120-minute SUV_peak_ (Fig. 2 and Supplementary Fig. S3)*.*

**Fig. 3:**
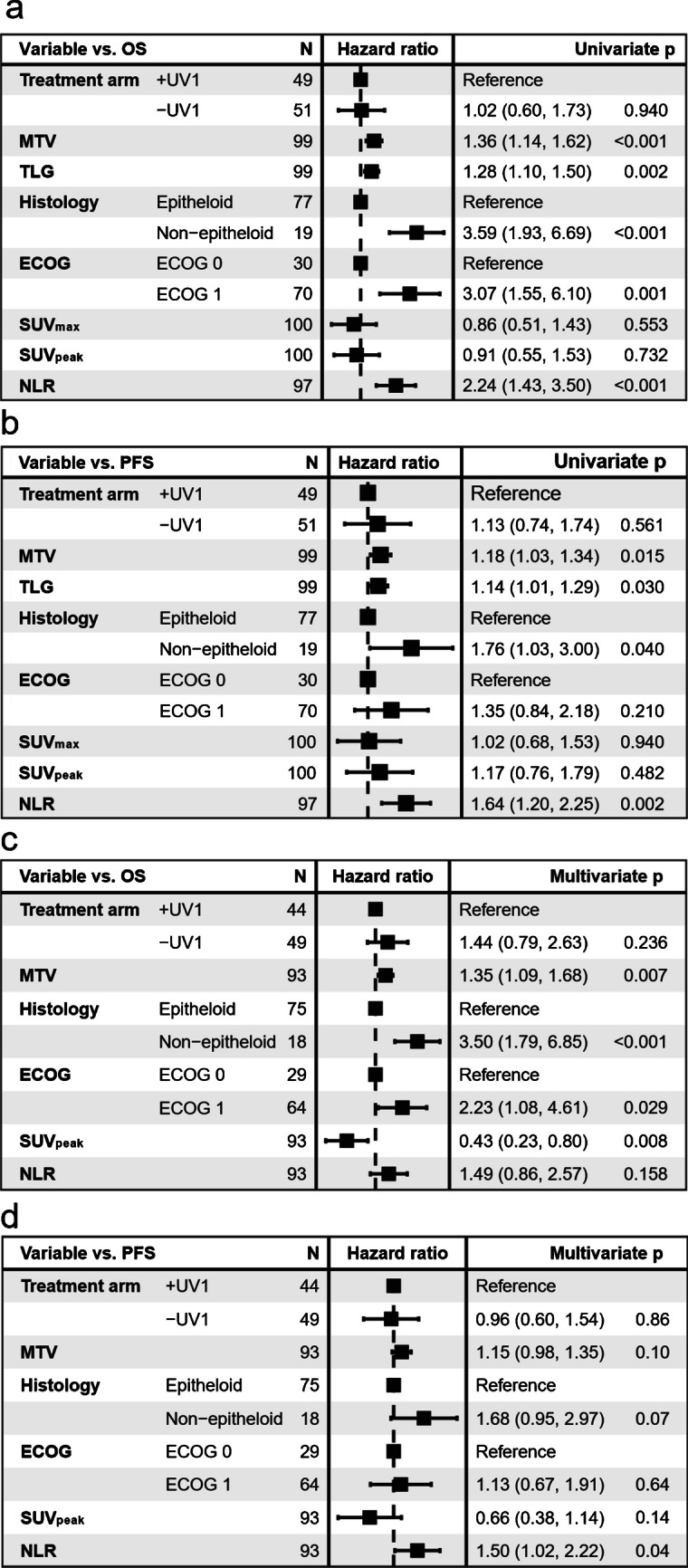
Univariate cox regression analyses with PET features from the baseline 60-minute scan for OS (**a**) and PFS (**b**), and multivariate Cox regression analyses for OS (**c**) and PFS (**d**). Continuous variables (MTV, TLG, SUV_max,_ SUV_peak_ and NLR) are log transformed. OS = Overall survival. PFS = Progression-free survival. ECOG = Eastern Cooperative Oncology Group performance status. UV1 = UV1 telomerase vaccine. MTV = metabolic tumour volume. TLG = total lesion glycolysis. SUV_max_ = maximum standardised uptake value. SUV_peak_ = peak standardised uptake value. NLR = neutrophil-to-lymphocyte-ratio. Four patients are excluded in the multivariate analyses due to not available histologic status, three due to not available NLR and one due to unobtainable MTV delineation

In univariate Cox regression analysis, MTV from the 60- and 120-minute baseline scans showed significant associations with OS and PFS (Fig. 3 and supplementary Fig. S3). In multivariate Cox regression, MTV from the baseline 60-minute scan was significantly associated with OS, but it did not show a significant association with PFS. MTV from the 120-minute baseline scan was not significantly associated with OS, however, it was significantly associated with PFS (Fig. 3 and supplementary Fig. S4).

Neither the SUV_peak_ from the 60-minute nor 120-minute baseline scans demonstrated significant associations with OS or PFS in univariate analyses. In multivariate analyses, it was significantly associated with OS at the 60-minute baseline scan. (Fig. 3 and supplementary Fig. S4).

Cox regression analyses for TLG and SUV_max_ showed comparable results to MTV and SUV_peak_ (Fig. 3 and Supplementary Fig. S5).

In univariate analysis, non-epithelioid histology, NLR, and ECOG performance showed significant associations with OS. In the multivariate analysis, only histology and ECOG emerged as significant predictors of OS. Similarly, for PFS, non-epithelioid histology and NLR were significantly associated in univariate analysis. In the multivariate analysis, significant associations with PFS were only observed for NLR (Fig. 3).

**Fig. 4:**
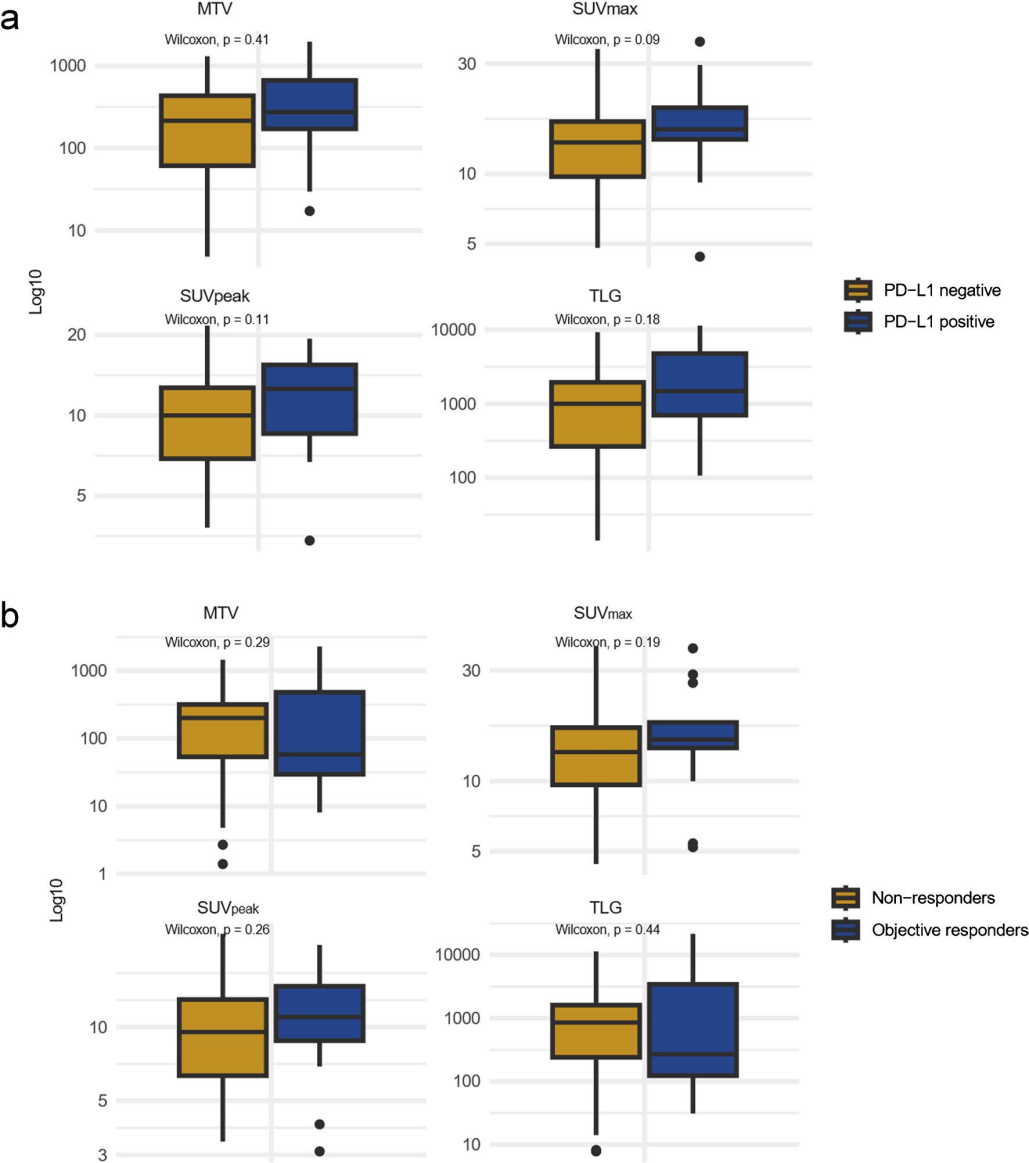
Associations between PET features from the baseline 60-minute scan with PD-L1 and treatment response. **a**) Associations between PD-L1 status and PET features from the baseline 60-minute scan, where patients are divided into PD-L1 positive (> 1) and PD-L1 negative (< 1). **b**) PET features from the baseline 60-minute scan in patients with an objective response vs. patients without an objective response. Objective responders = patients with partial response according to modified Response Criteria in Solid Tumours (mRECIST) and immune RECIST (iRECIST). Non-responders = patients with stable disease (SD) or progressive disease (PD) as their best overall response according to mRECIST and iRECIST. PD-L1 = programmed death ligand-1. PET = positron emission tomography. MTV = metabolic tumour volume. TLG = total lesion glycolysis. SUV_max_ = maximum standardised uptake value. SUV_peak_ = peak standardised uptake value

No significant differences in MTV, TLG, SUV_max_ or SUV_peak_ were found between PD-L1 positive and PD-L1 negative in the baseline 60-minute scan nor the baseline 120-minute scan (Fig. 4. and Supplementary Fig. S6).

There was no significant difference between objective responders and non-responders for baseline 60-minute MTV, TLG, SUV_max_, or SUV_peak_ (Fig. 4).

**Fig. 5:**
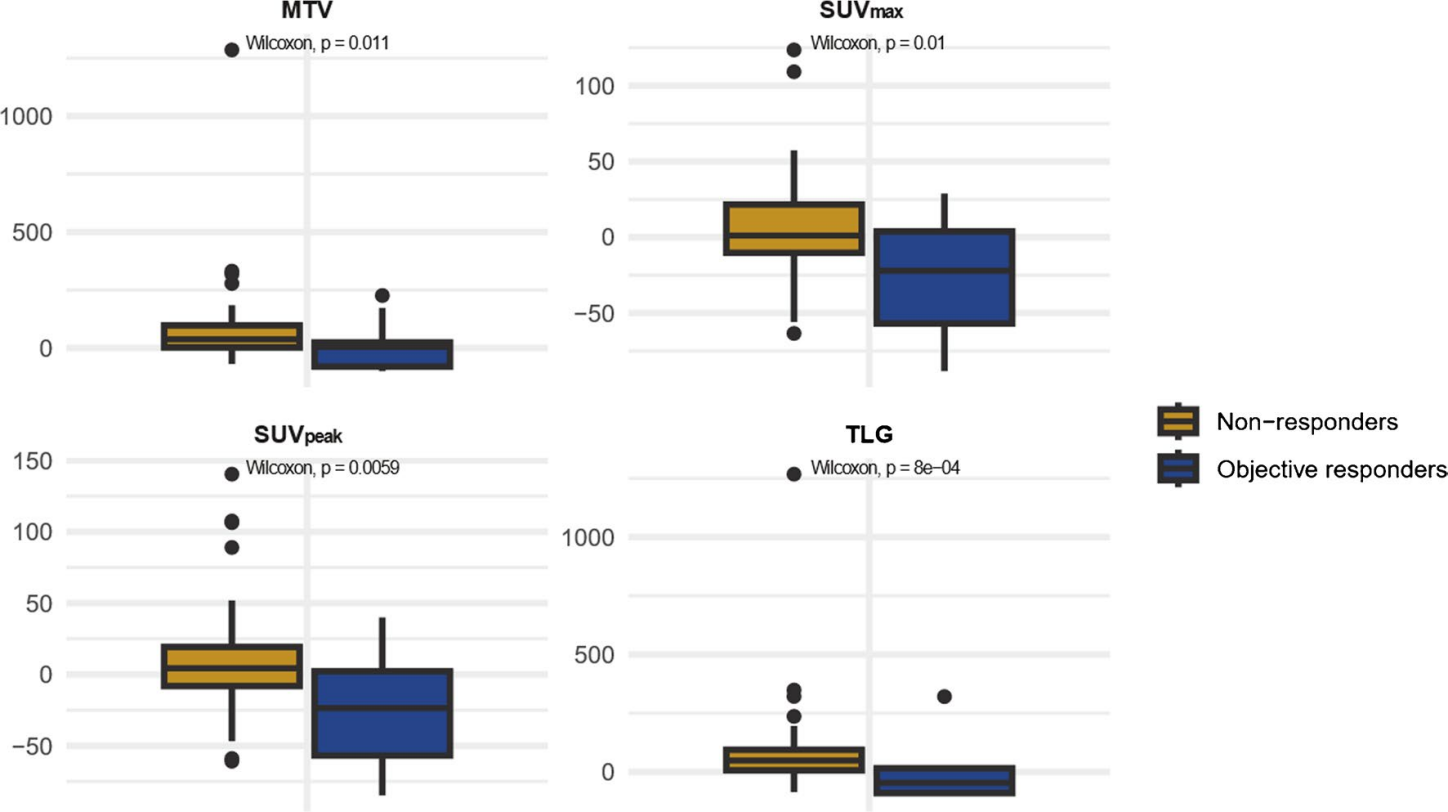
Changes in PET features from baseline to week-5. Illustration of the changes in PET features from baseline to week-5 in patients with an objective response versus non-responders (consisting of stable disease and progressive disease ) according to mRECIST and iRECIST. mRECIST = modified Response Criteria in Solid Tumours (mRECIST). iRECIST = immune RECIST. MTV = metabolic tumour volume. TLG = total lesion glycolysis. SUV_max_ = maximum standardised uptake value. SUV_peak_ = peak standardised uptake value

There was a significant difference in the changes in MTV, TLG, SUV_max_ and SUV_peak_ from baseline to week-5 among objective responders compared to non-responders (p = 0.01, p < 0.001, p = 0.01 and p = 0.006, respectively). The median decline in TLG for objective responders was -47% (Q1 -57 %, Q3 6 %), whereas non-responders showed a median increase of 49 % (Q1 12 %, Q3 97 %). Objective responders had a median decline in SUV_max_ of -22 % (Q1 -57 %, Q3 4 %), compared to a median increase of 4 % (Q1 -10 %, Q3 22 %) observed in non-responders. For SUV_peak_, the median decline among objective responders was -24 % (Q1 -57 %, Q3 2 %), while non-responders experienced a median increase of 4 % (Q1 -8 %, Q3 19 %). The median MTV increased by 4 % (Q1 -80 %, Q3 24 %) in objective responders, compared to a median increase of 37 % (Q1 2 %, Q3 97 %) in non-responders. (Fig. 5)

## Discussion

In our study, MTV from the baseline 60-minute scan was significantly associated with OS and PFS in univariate and with OS in multivariate Cox regression analysis. Although not statistically significant, a similar trend was observed for PFS in multivariate analysis. Patients exhibiting an objective treatment response had a significant decline in TLG, SUV_max_ and SUV_peak_ at week-5 compared to non-responders.

The association between survival outcome and non-epitheliod histology, ECOG performance status and NLR aligns with existing literature [28, 29, 30, 31, 32, 33].

Tumour volume is a well-documented prognostic indicator in malignancies [34–36]. MTV is a measure of metabolically active tumour volume, while TLG is traditionally understood as a measure of glycolytic burden in the tumour. Existing studies examining PET characteristics in PM have often demonstrated a significant relationship with both TLG and SUV_max_ and survival outcome, while results for MTV have been more variable –suggesting SUV to be a more robust prognostic indicator than volume [10–12, 37]. However, we found MTV and TLG to exhibit nearly identical associations with outcomes (Fig. 3 and supplementary Fig S5). This aligns with Reynolds et al.’s findings [38], which, after investigating MTV and TLG in patients with PM undergoing immuno-chemotherapy, argued that TLG serves more as a volume rather than a glycolytic activity indicator. They concluded that SUV_mean_ is of inferior importance to TLG due to the high interpatient variance in MTV compared to a more constant SUV_mean_, and that for PM both MTV and TLG are a measure of volume [38]. The study by Reynolds et al. is limited by its small sample size and it did not explore other SUV metrics like SUV_max_ and SUV_peak_. In contrast, our analysis evaluated SUV_max_ and SUV_peak_, without examining SUV_mean_. Nevertheless, in our data, the correlation between MTV and TLG was 0.97 (p < 0.0001) at the baseline 60-minute scan and 0.98 (p < 0.0001) at the week-5 scan, supporting the view that TLG primarily indicates volume. Consequently, it may be feasible to opt only for MTV.

Aligned with research showing that high tumour burden may indicate effective immune evasion [39], our findings revealed that high MTV was associated with inferior outcomes. Interestingly, no significant difference in baseline MTV was found between non-responders and objective responders, illustrating that an objective treatment response was also observed in patients with high tumour burden. However, the sub-analysis including only EARL2 images showed a significantly lower MTV among the objective responders compared to the non-responders (supplementary Fig. S7). Upon further exploration, we found that this difference in result was explained by several patients with high baseline tumour volume from one study site who experienced an objective response. More studies are needed to investigate whether a high tumour burden affects the efficacy of immunotherapy in PM.

**Fig. 6:**
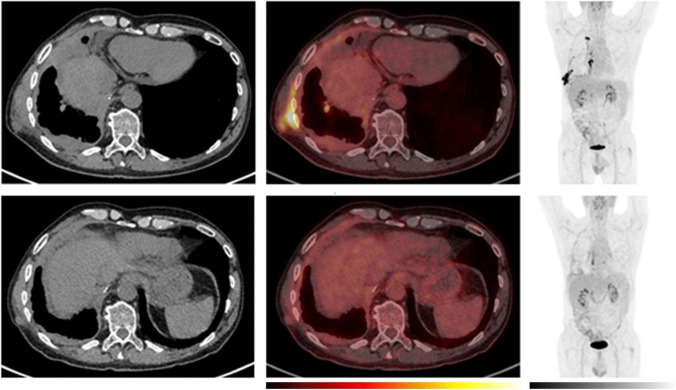
Example of a patient with a decline in MTV and [^18^F]fluorodeoxyglucose (FDG) uptake as early as the week-5 PET scan which was followed by a long-term objective tumour response. Comparative imaging from baseline (top row) and week-5 (bottom row). To the left, axial computer tomography (CT) images, in the middle, axial positron emission tomography (PET)/CT images and to the right the maximum intensity projection (MIP). The PET images are observed within a 0-10 SUV window. The patient was classified as having stable disease at the first CT response assessment, before developing partial response at the week-12 CT scan according to the modified Response Criteria in Solid Tumours (mRECIST) and immune RECIST. While the patient had stable disease at the week-5 CT scan, PET/CT already showed a reduction in metabolic tumour volume (MTV) and a decline in [^18^F]FDG uptake in the tumour. The treatment was discontinued after 7 months due to intolerable toxicity. The patient is still under follow-up 20 months after the start of the treatment

Patients with an objective response demonstrated a notable decline in both TLG and SUV metrics at the week-5 scan. For half of these patients, the objective response was not yet evident at the first CT response assessment, indicating the potential of PET scans in early response assessment in PM undergoing immunotherapy (Fig. 6).

**Fig. 7:**
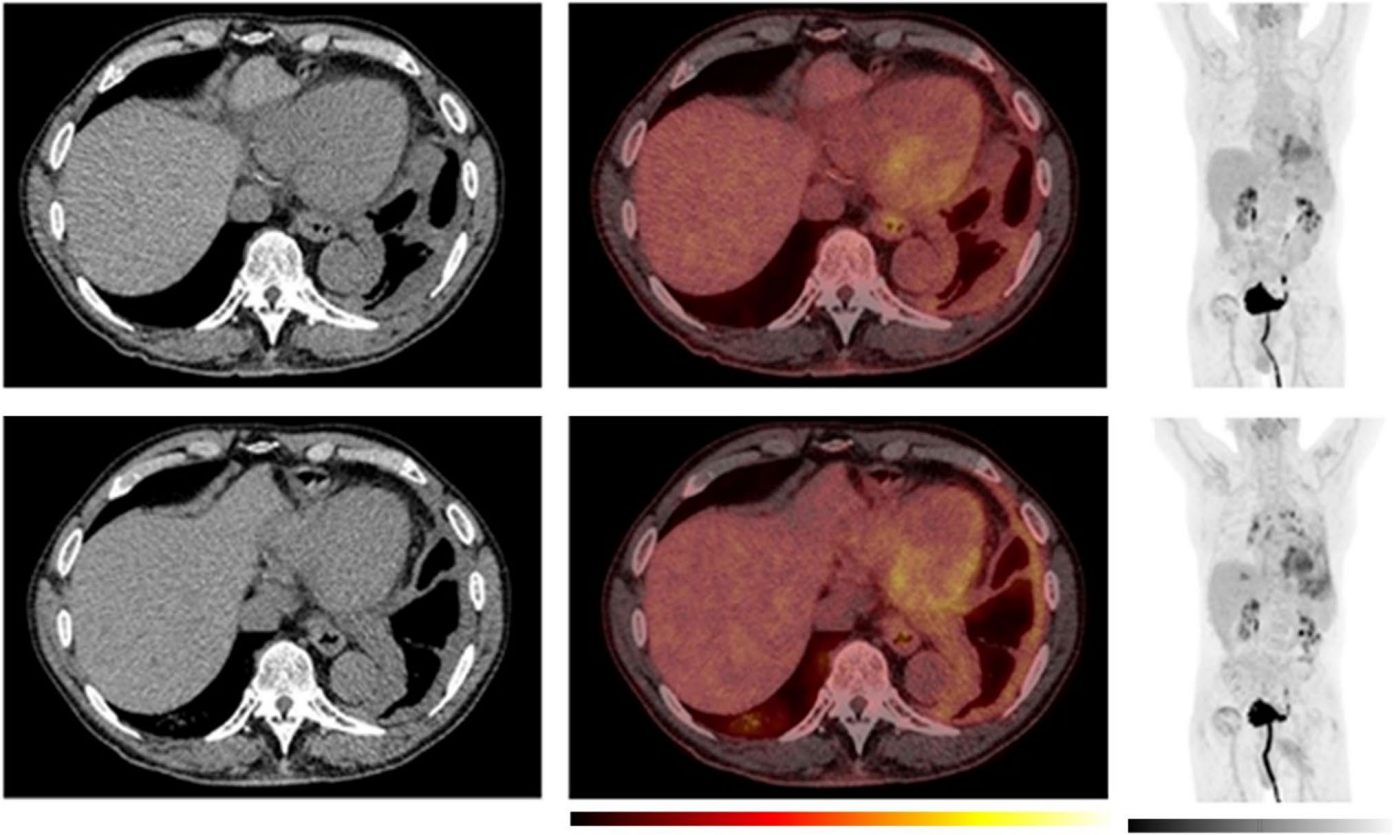
Example of a patient with an initial increase in MTV and [^18^F]fluorodeoxyglucose (FDG) uptake despite long-term objective tumour response/disease control. Baseline (top row) and week-5 (bottom row). To the left, axial computer tomography (CT) images, in the middle, axial positron emission tomography (PET)/CT images and to the right the maximum intensity projection (MIP). The PET images are observed within a 0-10 SUV window. While the patient had stable disease according to the modified Response Criteria in Solid Tumours (mRECIST) at the first CT response assessment, both metabolic tumour volume (MTV) and [^18^F]FDG uptake showed an increase from baseline to week-5. The patient was classified as having a partial response at the week-12 CT scan. The patient demonstrated clinical signs of improvement already at the week-5 visit. The planned one-year PET scan was cancelled due to hip surgery. The treatment was discontinued after 9 months due to the development of severe Immune-mediated thrombocytopenia (ITP) and the patient died of disease progression 2.5 years after the start of the treatment

**Fig. 8:**
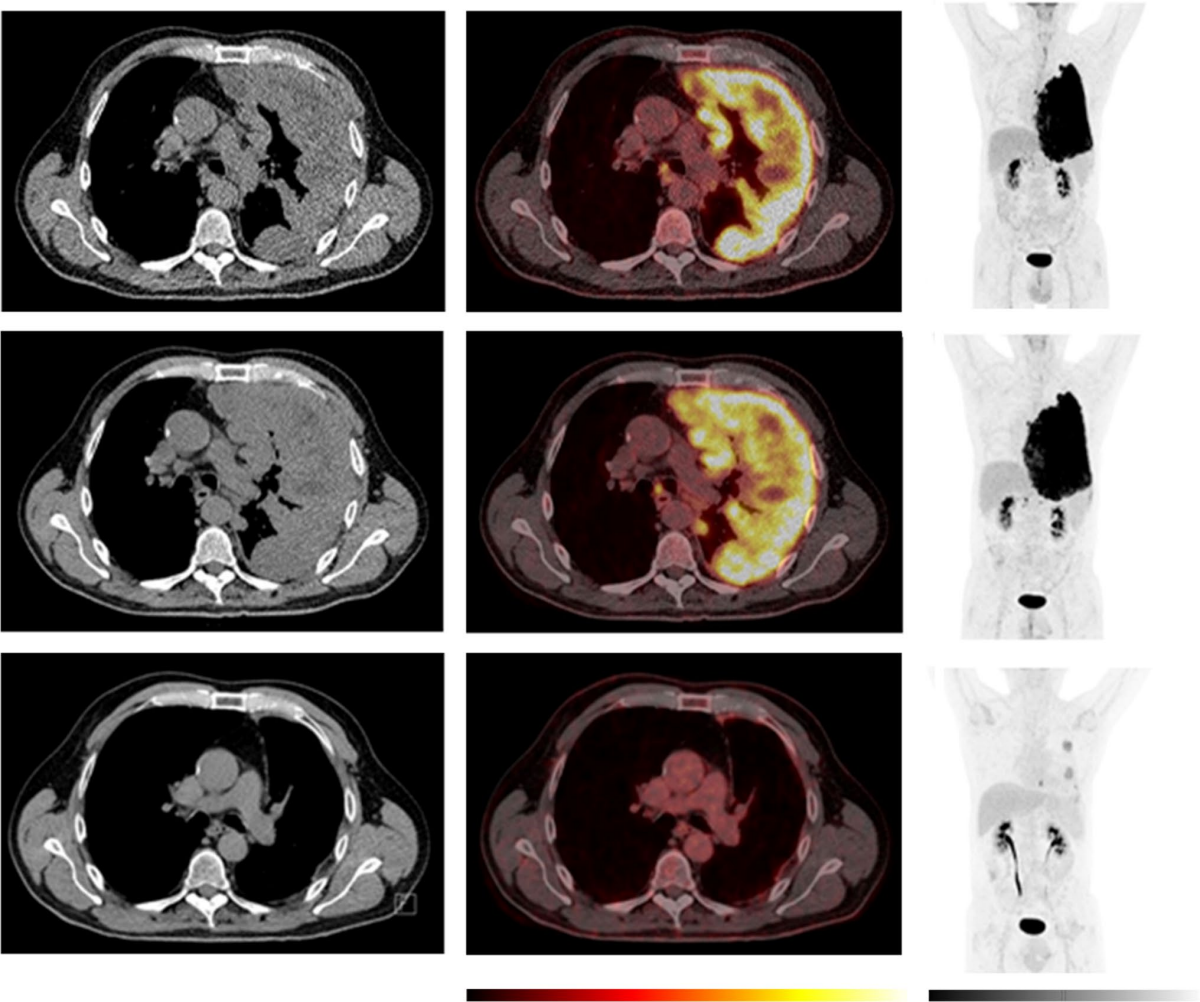
Example of a patient with a pseudoprogression. An initial increase in tumour volume and MTV and a decline in [^18^F]fluorodeoxyglucose (FDG) uptake at week-5 was followed by a long-term objective tumour response/disease control. Comparative imaging from baseline (top row), week-5 (middle row), and one-year (bottom row). To the left, axial computer tomography (CT) images, in the middle, axial positron emission tomography (PET)/CT images and to the right the maximum intensity projection (MIP). The PET images are observed within a 0-10 SUV window. The patient was classified as having progressive disease at the first CT response assessment, before developing stable disease at the week-12 and partial response at the week-18 CT scan according to the modified Response Criteria in Solid Tumours (mRECIST) and immune RECIST. At the week-5, the PET/CT showed an increase in metabolic tumour volume (MTV), while [^18^F]FDG uptake (SUV_max_ and SUV_peak_) has started to decline. At the one-year follow-up PET/CT, there is a notable change, with a visible reduction in tumour size, MTV, and [^18^F]FDG uptake. This corresponded with clinical performance status which was reduced at week-5 and improved from week 12 and onwards. The patient died due to disease progression 2 years after the start of the treatment

However, this was not uniform across all responding subjects. In a few responders, an increase in volumetric PET features and [18F]FDG uptake was observed at the week-5 PET scan (Fig. 7). Two patients experienced a pseudoprogression according to iRECIST, of whom one had a week-5 PET scan. Interestingly, while MTV increased in this patient, a decline in [18F]FDG uptake was observed at the week-5 scan (Fig. 8). This variability highlights the complexity of immunotherapy response mechanisms and underscores the need for further research.

In previous PM studies, SUV_max_ is often associated with a worse outcome, while for SUV_peak_ existing studies are limited [10–12, 37]. In our data, there was a high correlation between SUV_max_ and SUV_peak_. Furthermore, in the survival analyses, the results for SUV_max_ and SUV_peak_ were similar. High baseline

SUV_max_/SUV_peak_ in our study did not correlate with worse outcomes, rather the opposite was observed for high SUV_max_ and SUV_peak_ in the multivariate Cox regression analyses from the baseline 60-minute scan. This result is in contrast to the majority of previous studies, in which high SUV_max_ has been associated with a poorer outcome [10–12, 37]. Tests for multicollinearity and interaction effects between the variables were conducted without any explanatory findings. In previous studies, the patients were typically treated with chemotherapy rather than immunotherapy. Tumours exhibiting increased cell density and proliferation, which are often mirrored in increased [^18^F]FDG uptake [40, 41], generally correlate with more aggressive disease and poorer patient outcomes. However, the biological response mechanisms invoked by immunotherapy, which emphasise the immunological characteristics of the tumour and its’ microenvironment [42], might explain the lack of association between high SUV_max_/SUV_peak_ and poor outcomes in our study. Our findings indicate that the traditional negative prognostic value of high SUV_max_ observed in PM treated with chemotherapy might not apply in the context of immunotherapy, and rather, a contrary trend might be present.

PD-L1 expression is known to be a predictor of response to immunotherapy in several malignancies, although this is not uniform across all cancer types [43]. Although the exact mechanisms are still unknown, [^18^F]FDG uptake has been associated with the expression of PD-L1 and CD8+ tumour infiltrating lymphocytes (TILs) [44–47]. High PD-L1 expression is generally considered a poor prognostic indicator in PM, yet studies have shown conflicting results regarding the link between PD-L1 expression and immunotherapy response, summarised in a review by Perrino et. al [48]. We did not find a correlation between PD-L1 expression and PET features in the analysis of all baseline PET scans. However, in the sub-analysis of EARL2-compliant scans, there was a significantly higher MTV and TLG at the 60-minute baseline scan, as well as a significantly higher MTV, TLG, SUV_max_ and SUV_peak_ at the 120-minute baseline scan among PD-L1 positive compared to PD-L1 negative (supplementary Fig. S7 and Fig. S8). In the NIPU trial, the correlation between PD-L1 expression and treatment response was not examined due to a substantial number of patients with indeterminate PD-L1 status [49]. Consequently, caution is advised when interpreting the association between PET features and PD-L1 in our data. Further research is necessary to explore the potential relationship between SUV metrics and PD-L1 in PM, as well as the association between immunotherapy response and PD-L1 expression in this context.

[^18^F]FDG PET/CT features can offer valuable insights into individual disease trajectories, potentially guiding treatment decisions and contributing to personalised patient care in PM. To effectively use PET in outcome prediction and response assessment, standardised methods for image evaluation and tumour delineation are essential. Continued research and rapid advancements in artificial intelligence (AI) and radiomics hold the potential to transform the practical use of PET in PM [3, 50–53].

### Limitations

There are several limitations in this study. Primarily, the study sample size is limited. Additionally, a notable proportion of the patients with [18F]FDG PET/CT scans did not meet the EARL2 specifications. Therefore, a sub-analysis was conducted that included only EARL2-compliant images (for patient characteristics see supplementary Table S1). The result of the sub-analysis gave comparable results, with the exception of the comparison between PD-L1 status and PET features, and the comparison of baseline PET features between objective responders and non-responders (see supplementary Fig. S7 and Fig. S8). The discrepancy in results concerning PD-L1 status may be explained by a smaller fraction of patients with unknown PD-L1 status among patients with EARL2 compliant images. Moreover, the utilisation of a threshold-based delineation software presented challenges. The threshold was determined by the investigators through consensus and was based on a visual interpretation of the images, which may challenge the repeatability of the results. Other thresholds, such as fixed percentage thresholds, fixed SUV, and thresholds based on blood background were initially attempted but did not provide satisfactory delineations (Supplementary Fig. S1). A fixed threshold sometimes included the heart, mediastinal, or abdominal regions, while a fixed percentage of 40 % SUV_max_ often resulted in PM lesions being excluded. These challenges are closely related to the distinct pleural growth patterns, varying tumour thickness and SUV heterogeneity observed in PM, in contrast to focal lesions in other malignancies. In the prognostic results, the wide range of MTV (5 to > 2000 cm^3^) mitigates the impact of the delineation methodology. The comparison of MTV between baseline and follow-up remains methodologically challenging. However, the literature discourages the use of thresholds based on a fixed SUV or a fixed percentage of SUV_max_ when analysing volumetric PET metrics at various time points within the same patient due to changes in [18F]FDG uptake [54]. While our method is less vulnerable to changes in [18F] FDG uptake, caution should still be taken when interpreting changes in MTV from baseline to week-5.

## Conclusion

Our study finds MTV to be an outcome predictor in PM treated with immunotherapy. Contrary to previous PM studies where patients were treated with chemotherapy, our results do not suggest inferior outcomes in patients with high SUV_max_ or SUV_peak_, possibly due to the unique mechanisms of immunotherapy. An early reduction in TLG, SUV_max_ and SUV_peak_ was associated with an objective treatment response. While there was an association between early decline in TLG, SUV_max_ and SUV_peak,_ and objective treatment response, a minority displayed an initial increase in PET metrics before subsequently having a radiological and clinical response, which may reflect a different immune response. Further studies are warranted to investigate the associations between PET features, immune features of mesotheliomas and the response to immunotherapy.

## Supplementary Information

Below is the link to the electronic supplementary material.

Supplementary file1 (DOCX 4.00 MB)

## Data Availability

The datasets analysed during the current study are available from the corresponding author on reasonable request.
