# Supplementary material for: Outcome prediction based on [18F]FDG PET/CT in patients with pleural mesothelioma treated with ipilimumab and nivolumab +/- UV1 telomerase vaccine
Source: Eur J Nucl Med Mol Imaging. 2024 Aug 12;52(2):693–707. doi: 10.1007/s00259-024-06853-0 (PMC11732904; doi:10.1007/s00259-024-06853-0)
Supplement: Supplementary file 1 — Supplementary file1 (DOCX 4.00 MB) [file 259_2024_6853_MOESM1_ESM.docx]

**Outcome prediction based on [18F]FDG PET/CT in patients with pleural mesothelioma treated with ipilimumab and nivolumab +/- UV1 telomerase vaccine**

**Supplementary**


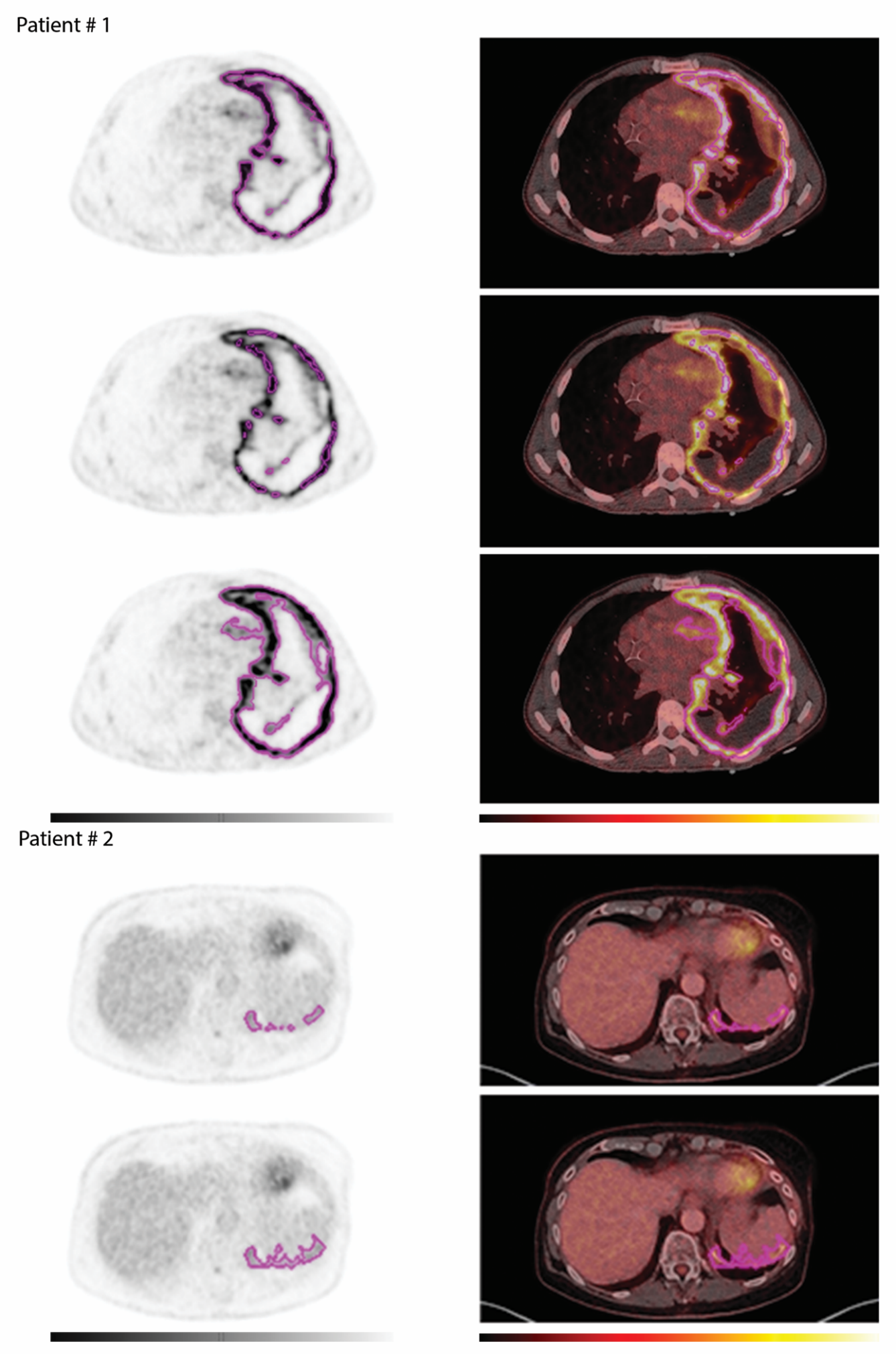


[**Fig. S1**](#Figure_S2) *Illustration of the delineation process based on various thresholds; a flexible percentage of SUV*_max_*, 40 % of SUV*_max_ *and blood-background. To the left axial PET images and to the right PET/CT images The PET images are observed within a 0-10 SUV window.* *Patient #1. Top row: The threshold was adjusted to 25 % of SUV*_max_ *to include tumour lesions with increased [18F] FDG uptake. Middle row: Threshold set as 40 % of SUV*_max_ *leading to the exclusion of areas with tumour tissue with increased [18F]FDG-uptake. Bottom row: Threshold based on blood background, resulting in the inclusion of areas around the tumour lesions, e.g. parts of the heart. Patient # 2. Top row: Threshold adjusted to 50 % of SUV*_max_ *as lower percentages included areas around the tumour lesions. Bottom row: Threshold set to 40 % of SUV*_max_ *leading to inclusion of areas around the tumour lesions, e.g. parts of the spleen. The threshold based on blood background did not delineate any tumour lesions at all in this patient. SUV*_max_ *= maximum standardised uptake value*


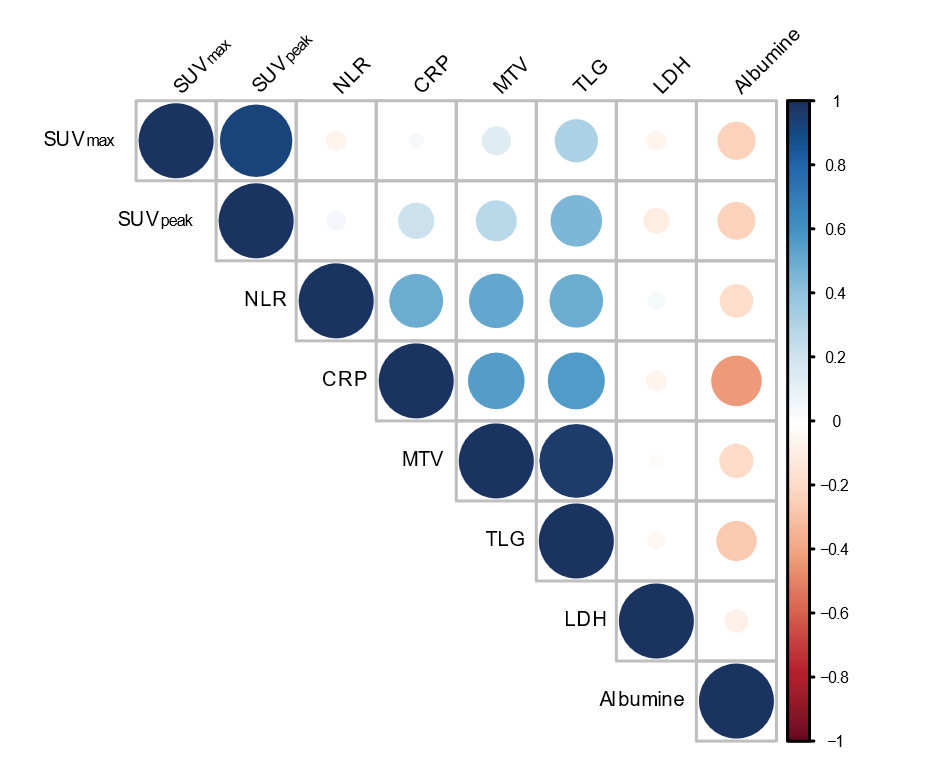


[**Fig. S**](#Figure_S1)**2** *Spearmanns correlation plot for SUV features from the baseline 60-minute scan and NLR, CRP, LDH and albumine taken at screening.* *UV1 = UV1 vaccine. MTV = metabolic tumour volume. TLG = total lesion glycolysis. SUV*_max_ *= maximum standardised uptake value. SUV*_peak_ *= peak standardised uptake value. NLR = neutrophil-to-lymphocyte-ratio. CRP = C-reactive protein. LDH = Lactate dehydrogenase*

**
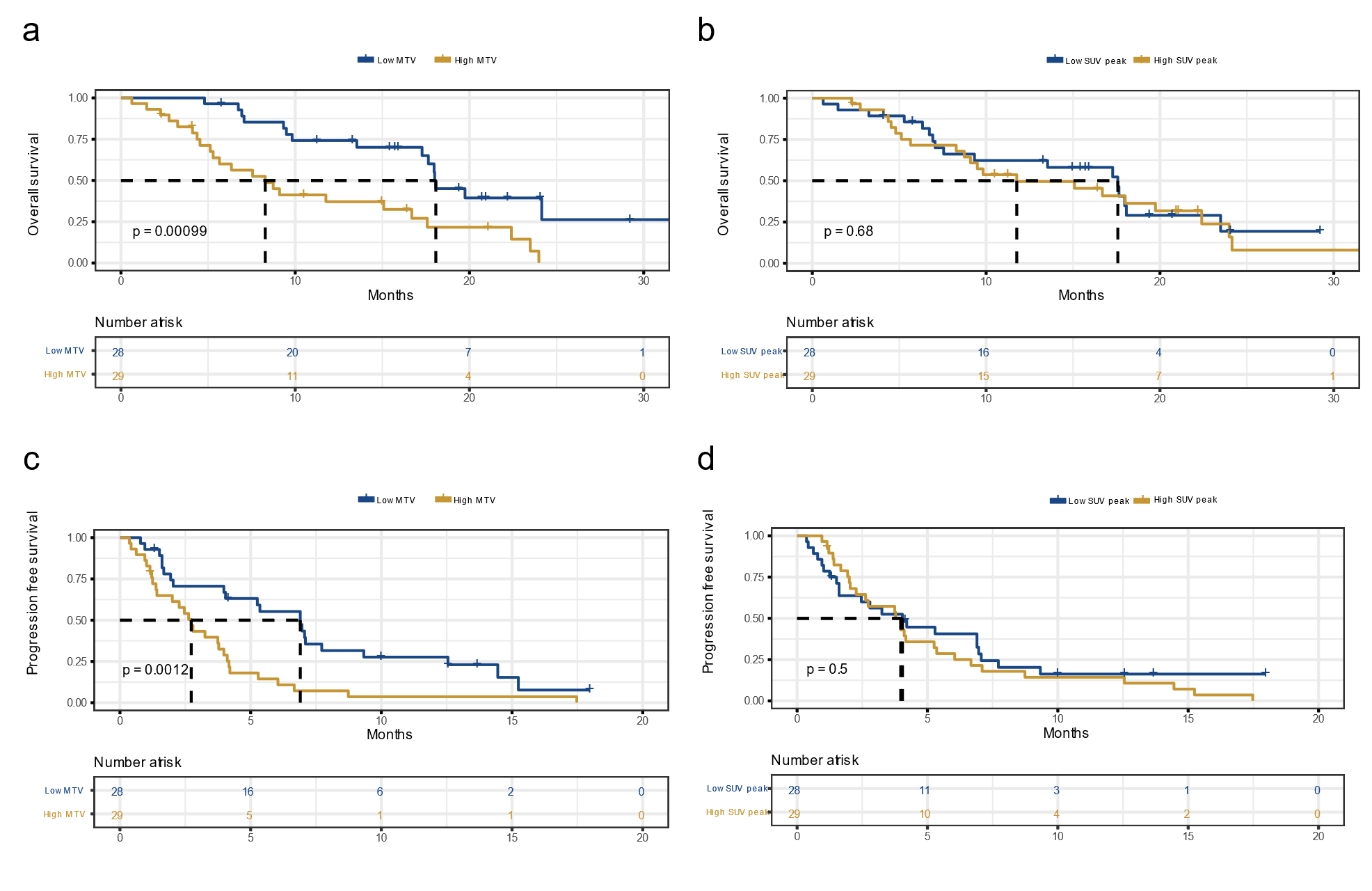
**[**Fig. S3**](#Figure_S3) *Kaplan Meyer curves with log-rank test based on MTV and SUV*_peak_ *from the baseline 120-minute scan. Panels a) and c) illustrate overall survival (OS) and progression-free survival (PFS), respectively where Low MTV and High MTV are grouped based on values below or above the median MTV. Panels b) and d) illustrate OS and PFS, respectively, where Low SUV*_peak_ *and High SUV*_peak_ *are grouped based on values below or above the median SUV*_peak_*.* *MTV = metabolic tumour volume. SUV*_peak_ *= peak standardised uptake value*


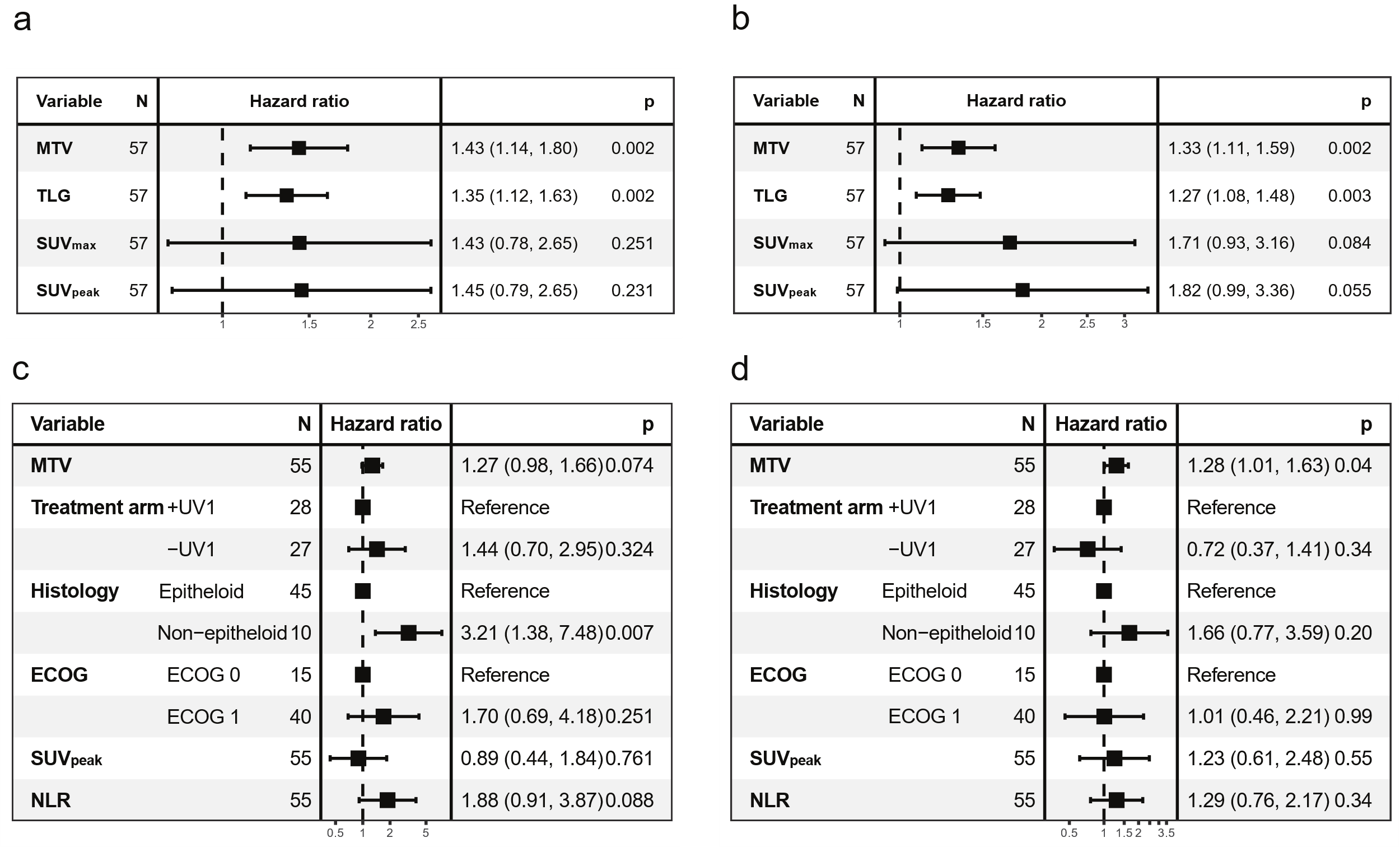


**[Fig. S4](#Figure_S4)** *Cox-models from the baseline 120-minute scan. a) and B) b Cox regression from the baseline 120-minute scan for OS and PFS, respectively. c) and d) multivariate Cox regression with MTV and SUV*_peak_ *for OS and PFS, respectively. OS = overall survival. PFS = progression free survival. Continuous variables (MTV, TLG, SUV*_max_*, SUV*_peak_ *and NLR) are in a log transformed scale. ECOG = Eastern Cooperative Oncology Group performance status. MTV = metabolic tumour volume. TLG = total lesion glycolysis. SUV*_max_ *= maximum standardised uptake value. SUV*_peak_ *= peak standardised uptake value. NLR = neutrophil-to-lymphocyte-ratio*

**
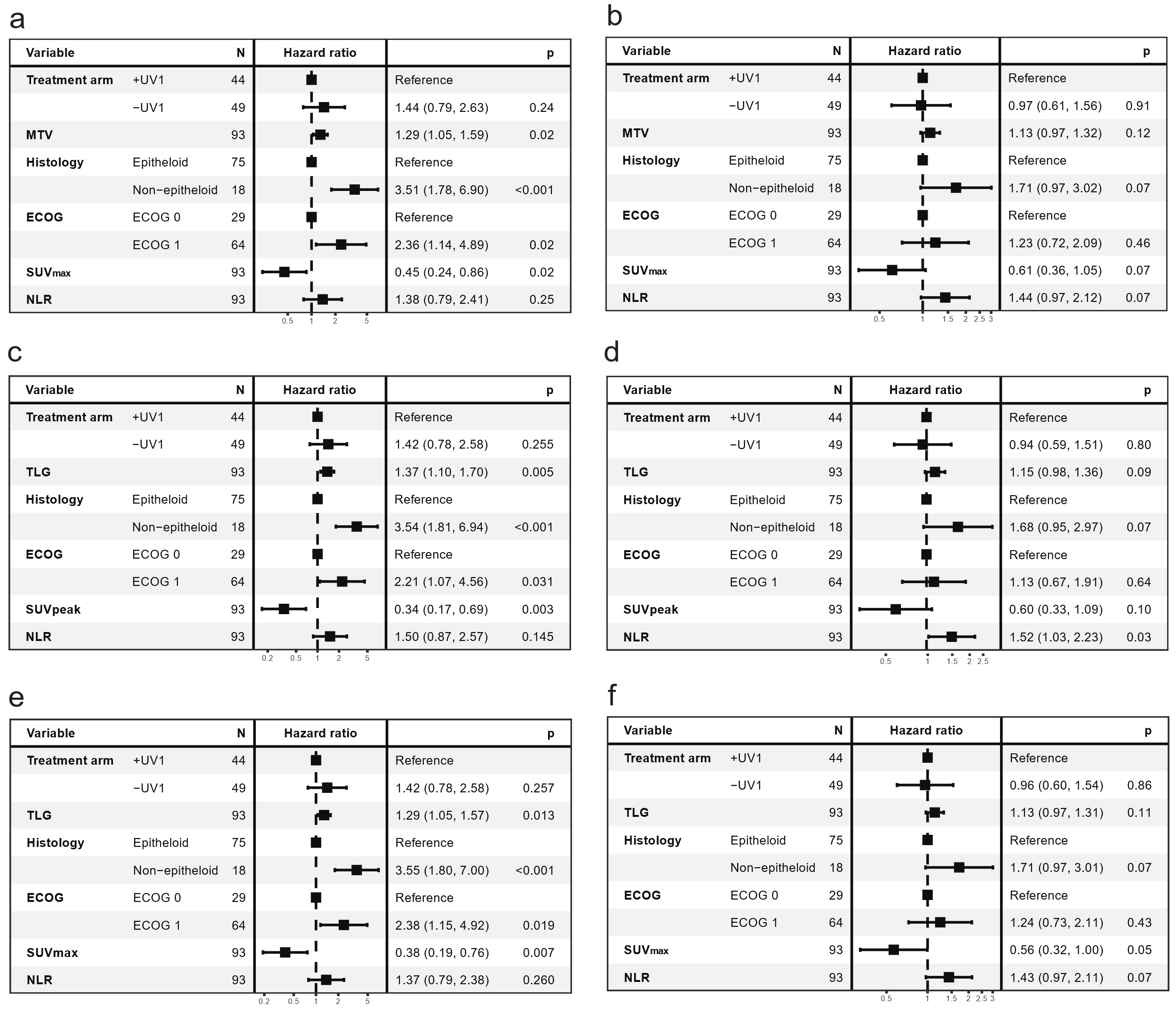
**[**Fig. S5**](#Figure_S6) *Multivariate Cox models from the baseline 60-minute scan. a) and b) multivariate Cox regression analysis for OS and PFS, respectively with SUV_max_ and MTV. c) and d) multivariate Cox regression analysis for OS and PFS, respectively, with SUV_peax_ and TLG. e) and f) multivariate Cox regression analysis for OS and PFS, respectively, with SUV_max_ and TLG. Continuous variables (TLG, MTV, SUV_max_, SUV_peax_ and NLR) are in a log-transformed scale. ECOG = Eastern Cooperative Oncology Group performance status. OS = Overall survival. PFS = progression-free survival. MTV = metabolic tumour volume. TLG = total lesion glycolysis. SUV_max_ = maximum standardised uptake value. SUV_peax_= peak standardised uptake value. NLR = neutrophil-to-lymphocyte-ratio*

**
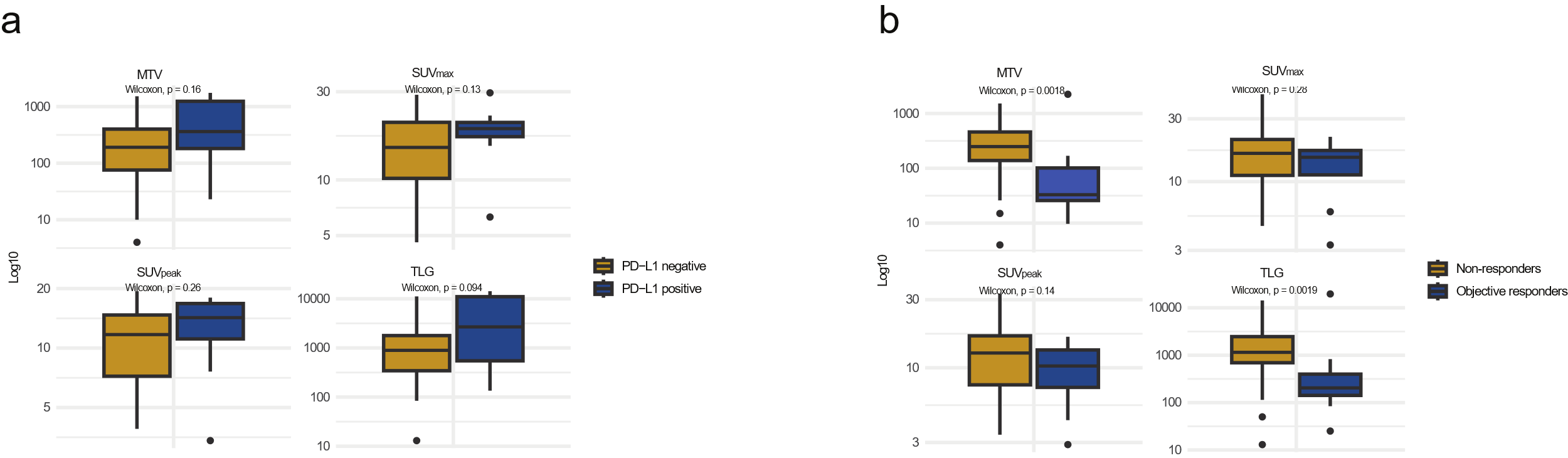
**[***Fig. S***](#Figure_S7)***6*** *Associations between PET features from the baseline 120-minute scan with PD-L1 (a) and treatment response (b). a) Associations between PD-L1 status and PET features from the 120-minute baseline scan, where patients are divided into PD-L1 positive (> 1) and PD-L1 negative (< 1). b) PET features from the 120-minute baseline scan in patients with an objective response vs. patients without an objective response. Objective responders = patients with partial response according to modified Response Criteria in Solid Tumours (mRECIST) and immune RECIST (iRECIST) as their best overall response. Non-responders = patients with stable disease (SD) or progressive disease (PD) according to mRECIST and iRECIST as their best overall response. PD-L1 = programmed death ligand-1. PET = positron emission tomography. MTV = metabolic tumour volume. TLG = total lesion glycolysis. SUV_max_ = maximum standardised uptake value. SUV_peak_ = peak standardised uptake value*

*
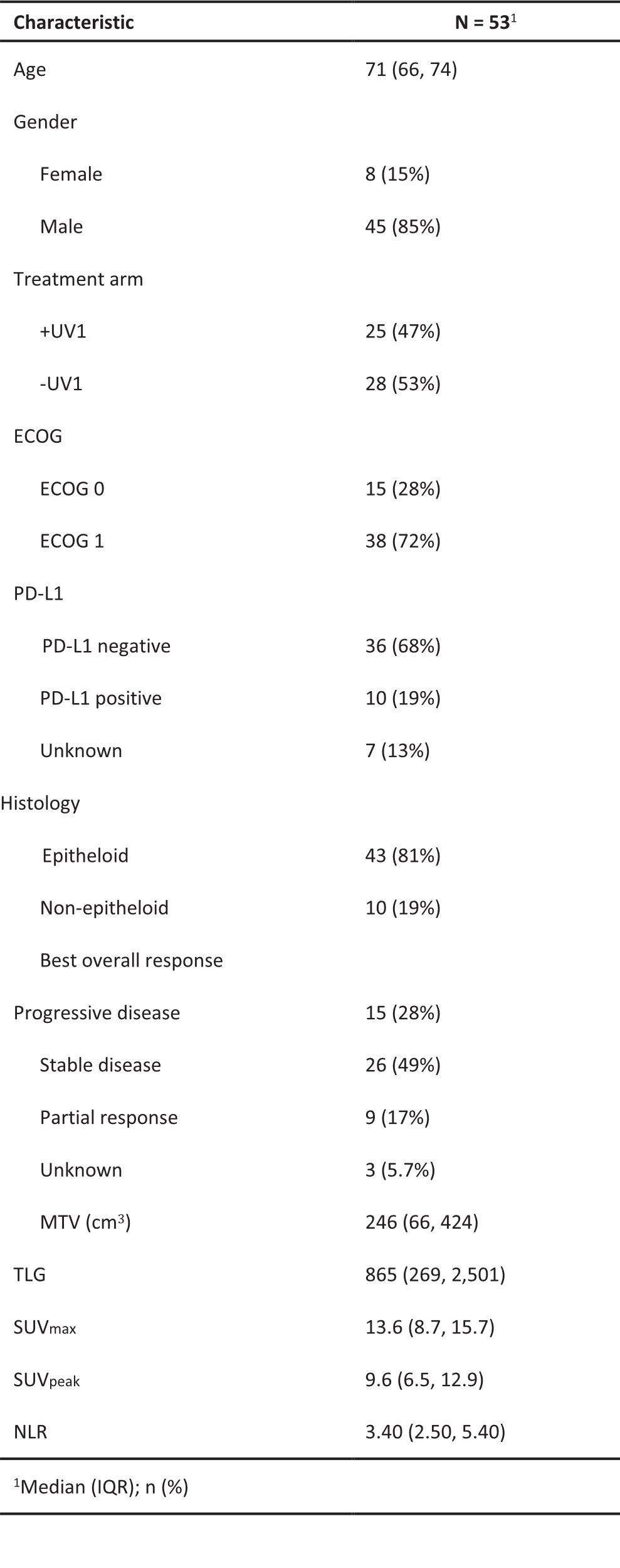
***[Table S1](#Tab_S1)** *Baseline characteristics of patients with EARL2 compliant PET scans. EARL = European Association of Nuclear Medicine Research Ltd. PET (positron emission tomography) features from the baseline 60-minute scan. UV1 = UV1 telomerase vaccine. ECOG = Eastern Cooperative Oncology Group performance status. Best overall response according to mRECIST (modified Response Criteria in Solid Tumours) and iRECIST (immune RECIST). MTV = metabolic tumour volume. TLG = total lesion glycolysis. SUV_max_ = maximum standardised uptake value. SUV_peak_ = peak standardised uptake value. NLR = neutrophil-to-lymphocyte-ratio*


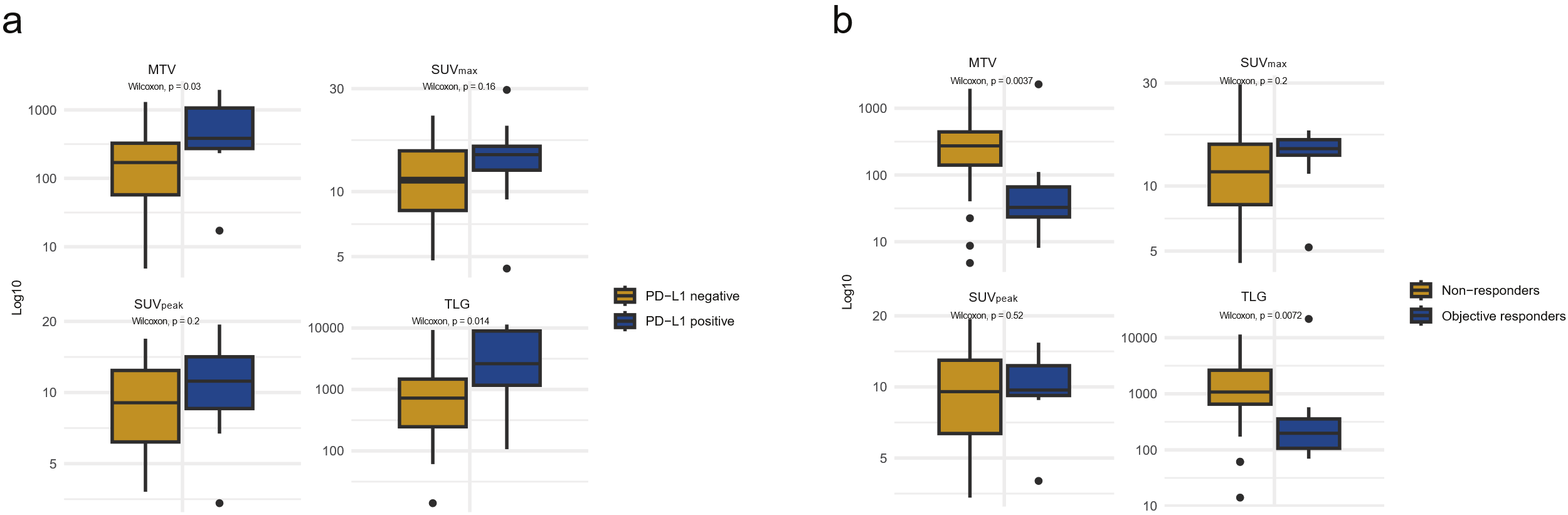


[**Fig. S**](#Figure_S7)**7** *Associations between PET features from EARL2 accreditated baseline 60-minute scans with PD-L1 (a) and objective treatment response (b). a) Associations between PD-L1 status and PET features from EARL2 accreditated 60-minute baseline scans, where patients are divided into PD-L1 positive (> 1) and PD-L1 negative (< 1). b) PET features from EARL2 accreditated 60-minute baseline scans in patients with an objective response vs. patients without an objective response. Objective responders = patients with partial response according to modified Response Criteria in Solid Tumours (mRECIST) and immune RECIST (iRECIST) as their best overall response. Non-responders = patients with stable disease (SD) or progressive disease (PD) according to mRECIST and iRECIST as their best overall response. EARL = European Association of Nuclear Medicine* *Research Ltd.* *PD-L1 = programmed death ligand-1. PET = positron emission tomography. MTV = metabolic tumour volume. TLG = total lesion glycolysis. SUV_max_ = maximum standardised uptake value. SUV_peak_  = peak standardised uptake value*

**
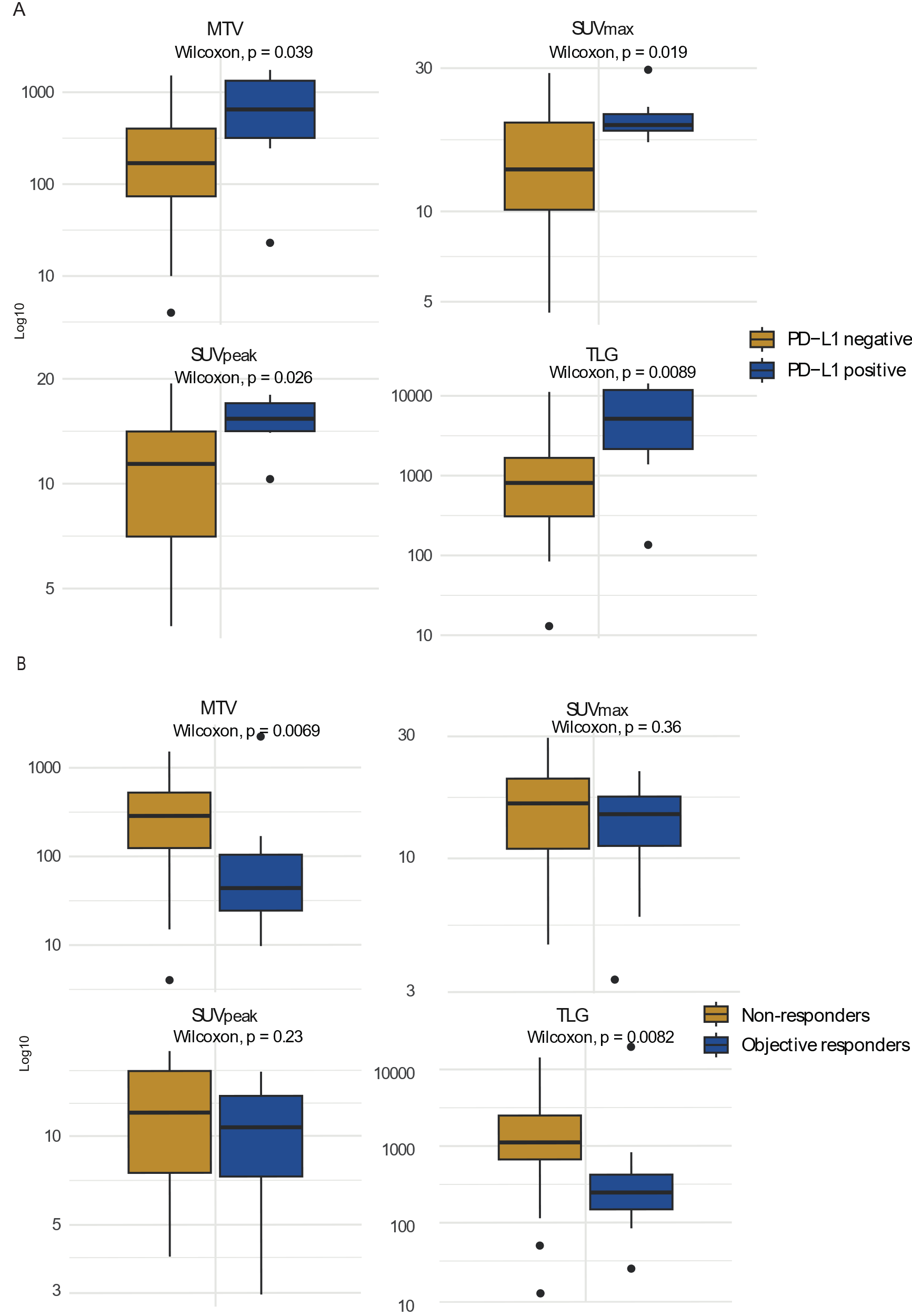
**[**Fig. S8**](#Fig_S8) *Associations between PET features from EARL2 accreditated baseline 120-minute scans with PD-L1 (a) and objective treatment response (b). a) Associations between PD-L1 status and PET features from EARL2 accreditated 120-minute baseline scans, where patients are divided into PD-L1 positive (> 1) and PD-L1 negative (< 1). b) PET features from EARL2 accreditated 120-minute baseline scans in patients with an objective response vs. patients without an objective response. Objective response = patients with partial response according to modified Response Criteria in Solid Tumours (mRECIST) and immune RECIST (iRECIST) as their best overall response. Non-responders = patients with stable disease (SD) and progressive disease (PD) according to mRECIST and iRECIST as their best overall response. EARL = European Association of Nuclear Medicine* *Research Ltd.* *PD-L1 = programmed death ligand-1. PET = positron emission tomography. MTV = metabolic tumour volume. TLG = total lesion glycolysis. SUV_max_ = maximum standardised uptake value. SUV_peak_ = peak standardised uptake value*
